# Integrating Metabolomics and Network Pharmacology to Decipher the Hepatoprotective Effect Mechanisms of Magnesium Isoglycyrrhizinate Injection

**DOI:** 10.3390/cimb46010019

**Published:** 2023-12-29

**Authors:** Yihua Zhang, Hui Li, Xueli Liu, Qiang Wang, Dong Zhao, Ming Su, Zhixin Jia, Shigang Shen

**Affiliations:** 1 Key Laboratory of Analytical Science and Technology of Hebei Province, College of Chemistry and Materials Science, Hebei University, Baoding 071002, China; zhangyihua0915@163.com (Y.Z.); lihui7171@163.com (H.L.); suming@hbu.edu.cn (M.S.); 2 NDMA Key Laboratory for Quality Control and Evaluation of Generic Drug, Hebei Institute for Drug and Medical Device Control, Shijiazhuang 050200, China; xueli2019@hotmail.com (X.L.); wqhky@163.com (Q.W.); zhaodong2016@126.com (D.Z.); 3 National Institutes for Food and Drug Control, Beijing 102629, China; jessiejzx@163.com

**Keywords:** magnesium isoglycyrrhizinate injection, liver injury, metabolomics, potential biomarker, network pharmacology

## Abstract

This study aimed to explore the liver protective effects of a fourth-generation glycyrrhizic acid product (magnesium isoglycyrrhizinate injection, MII) in the treatment of mice with drug-induced liver injury—specifically, to determine its effects on plasma metabolites. Moreover, the possible mechanism of its intervention in lipid metabolism and amino acid metabolism through the liver protective effect was preliminarily explored, combined with network pharmacology. The liver injury model of mice was established using acetaminophen (APAP). The protective effect of MII on the mice model was evaluated using pathological tissue sections and biochemical indices such as alanine transaminase (ALT), aspartate aminotransferase (AST), and superoxide dismutase (SOD). Metabolomics analysis of plasma was performed using the UHPLC-QTOF/MS technique to screen for potential biomarkers and enriched metabolic pathways. The potential targets and pathways of MII were predicted by network pharmacology, and the mechanism was verified by Western blot analysis. MII significantly improved the pathological liver changes in mice with liver injury. The content of ALT and AST was decreased, and the activity of SOD was increased significantly (*p* < 0.05, 0.01). A total of 29 potential biomarkers were identified in the metabolomics analysis, mainly involving seven pathways, such as lipid metabolism and amino acid metabolism. A total of 44 intersection targets of MII in the treatment of liver injury were obtained by network pharmacology, involving lipid metabolism and other related pathways. Western blot analysis results showed that MII could significantly reduce the expression of JAK2 and STAT3. MII can effectively ameliorate liver injury in modeled mice through related pathways such as lipid metabolism and amino acid metabolism. This study could provide not only a scientific basis for the elucidation of the mechanism of action of MII in exerting a hepatoprotective effect, but also a reference for its rational clinical application.

## 1. Introduction

The liver is the most important organ for drug metabolism [1]. Liver disease is a significant source of morbidity in humans and has become a significant global economic burden [2]. Meanwhile, the liver is also the main target organ for many harmful substances [3]. Drug-induced liver injury (DILI) is the main cause of acute liver injury, failure or even death [4]. Acetaminophen (APAP) has long been used as a fever reducer and pain reliever. However, although APAP has been found to be relatively safe at low doses, it may cause liver toxicity when used in excess [5,6]. Therefore, an overdose of APAP is often used in the modeling of drug-induced liver injury. The characteristics of liver injury induced by APAP include hepatocyte necrosis caused by mitochondrial stress [7]. In addition, APAP can form n-acetyl-p-benzoquinoneimine (NAPQI) by the catalysis of the cytochrome P450 enzyme. Additionally, NAPQI can cause oxidative stress by depleting intracellular glutathione at high doses [8].

Glycyrrhizic acid (GA) is a widely used component that is extracted from licorice root [9]. GA has a variety of pharmacological activities, such as liver protection, anti-liver toxicity, and anti-viral, anti-inflammatory, anti-cancer, hypolipidemic and hypoglycemic effects [10,11,12,13,14,15,16,17]. There are two isomers of GA, named 18α, 20β-glycyrrhizinic acid (18α-GA) and 18β, 20β-glycyrrhizinic acid (18β-GA) [18]. They have different structural and pharmacological activities, of which 18α-GA has stronger anti-inflammatory and hepatoprotective effects [19,20]. Magnesium isoglycyrrhizinate injection (MII) is a fourth-generation GA product that has a single configuration of 18α-GA, with content of more than 98%. MII is a liver cell protective agent that has anti-inflammatory effects, protects the liver cell membrane and improves liver function [21]. Studies have found that MII inhibits the JAK2/STAT3 pathway and TGF-β1/Smad signaling pathway by increasing miR-375-3p and improves high-fructose-induced liver fibrosis in rats [22]. MII inhibits inflammation and protects liver function through the STAT3 pathway [23]. MII maintains the level of Nrf2 in the liver, protects the antioxidant defense system, reduces oxidative stress and prevents ATO-induced liver injury [24]. MII improves concanavalin-A-induced liver injury through the p38 and JNK MAPK pathways [25]. Although there have been some studies reported, the protective mechanism of MII on APAP-induced liver injury has not been considered. APAP is a common cause of drug-induced liver injury, and it is valuable to study the mechanism of MII to improve the liver injury induced by APAP. Based on the existing studies, the JAK2/STAT3, TGF-β1/Smad, p38 and JNK MAPK signaling pathways may be the potential mechanisms involved in liver protection, but the specific mechanisms remain to be further clarified.

In this study, we conducted a comprehensive metabolomics and network pharmacology study to elucidate the mechanism of MII in the improvement of APAP-induced liver injury. First, UHPLC-QTOF-MS untargeted metabolomics was performed to screen for biomarkers and related metabolic pathways associated with MII’s effects on liver injury. Then, the mechanism of MII in ameliorating liver injury was discussed by means of integrated network pharmacology. Finally, Western blot analysis was used for verification. These results provide reference data for the further study of the liver protective effect of MII and also provide scientific support for the expansion of its clinical application.

## 2. Materials and Methods

### 2.1. Instrument, Drugs, and Material

An Agilent iFunnel 6550 Q-TOF LC/MS (Agilent Technologies, Santa Clara, CA, USA); AU480 Automatic Biochemical Analyzer (Beckman Coulter Inc., Brea, CA, USA); AT201 Electronic Balance (Mettler Toledo, Mettler, Zurich, Switzerland); and a BX53 microscope (OLYMPUS Corporation, Shinjuku, Tokyo, Japan, Eyepiece type DP72) were used.

MII (10 mL: 50 mg, batch No. 221101104) was purchased from Jiangsu Chia Tai Tianqing Pharmaceutical Co., Ltd. (Lianyungang, China). Diammonium glycyrrhizinate injection (DGI) was purchased from Huarun Shuanghe Limin Pharmaceutical Co., Ltd. (Jinan, China). Acetaminophen injection (20 mL: 2 g, batch No. 20220101) was purchased from Sichuan Jishanzhijia Pharmaceutical Co., Ltd. (Chengdu, China). Methanol, acetonitrile, formic acid and H_2_O were all of liquid chromatography–mass spectroscopy (LC–MS) grade (Fairfield, OH, USA), while all the remaining reagents were of analytical grade.

### 2.2. Animals and Ethics Statement

Male C57BL/6N mice (18 ± 22 g, 6–8 weeks old) were purchased from SPF (Beijing) Biotechnology Co., Ltd. (Beijing, China). The mice were kept under the conditions of a natural light–dark cycle (22 ± 2 °C and 40–50% humidity) with ad libitum access to water and standard food. All animal procedures were in accordance with the Regulations of Experimental Animal Administration issued by the State Committee of Science and Technology of the People’s Republic of China. The animal ethics were approved by the Animal Care and Use Committee of the Beijing University of Chinese Medicine (approval number: BUCM-2023032101-1055).

The mice were kept for a week before the experiment. The mice were randomly divided into 6 groups, with 16 mice in each group: control group, model group, low-dose group (LD), medium-dose group (MD) and high-dose group (HD). The LD, MD and HD groups were given MII (i.p.) (the doses in the low-, medium- and high-dose groups were 15, 30 and 60 mg/kg, respectively; these represented 0.5, 1 and 2 times the clinical equivalent dose of MII, respectively) for 7 consecutive days (Day 1–7). The positive drug group was given DGI 22.5 mg/kg (clinical equivalent dose) for 7 consecutive days (Day 1–7). The control group was given the same volume of normal saline. On Day 6, APAP (400 mg/kg) was injected intraperitoneally in the rats of the model, administration and positive drug groups [26].

### 2.3. Sample Collection

Serum, plasma and tissue samples were collected on the seventh day. Blood was collected by eyeball extraction in EP tubes with an anticoagulant. Plasma was obtained after centrifugation. Serum was obtained in EP tubes without an anticoagulant. Serum was used for biochemical indices detection and plasma was used for metabolomics analysis. Then, the mice were sacrificed by cervical dislocation, and the livers were removed to observe the morphology. The largest lobe was taken and fixed in paraformaldehyde for histopathological analysis, and the remaining part was frozen for storage.

### 2.4. Biochemical Index Detection and Histopathology

The content of AST, ALT and SOD in the serum of mice was determined by an automatic biochemical analyzer. The target organ, the liver, was stained by hematoxylin–eosin (HE) staining, and the effects of the drug intervention on the mouse liver before and after modeling were observed. After dehydration, embedding (paraffin), sectioning (5 μm) and staining, the liver fixed with paraformaldehyde was observed under an optical microscope by blinded personnel, and 6 views were observed; then, the main parts were photographed. According to the Suzuki score, the pathological scoring of liver injury was performed. The specific scoring criteria were as follows: score 0, no injury, normal liver parenchyma and interstitium; score 1, mild lesions, slight congestion in the hepatic sinus space, minor vacuole formation and single hepatocyte necrosis; score 2, the hepatic sinus space was mildly congested, a few vacuoles were formed, and less than 30% hepatocytes were necrotic; score 3, moderate injury, moderate hyperemia in the hepatic sinus space, moderate vacuole formation, about 30% to 60% hepatocyte necrosis; score 4, severe injury, severe congestion in the hepatic sinus space, severe vacuole formation, more than 60% of liver cell necrosis [27].

### 2.5. Metabolomics Analysis

#### 2.5.1. Sample Preparation

For each plasma sample, 100 μL of plasma was added to 300 μL of cold acetonitrile and vortexed for 30 s to precipitate proteins. The samples were centrifuged at 13,000 rpm for 10 min at 4 °C, and the supernatant was concentrated in a vacuum until dry. A total of 100 μL 10% acetonitrile solution was added and dissolved ultrasonically, and then centrifuged at 4 °C for 10 min at 13,000 rpm, and the supernatant was used for analysis. A total of 10 μL of plasma from each sample of the control group, model group and administration groups was uniformly mixed to obtain mixed plasma samples, which were recorded as quality control (QC) samples. The pretreatment method of QC samples was the same as that of plasma samples. The analysis of QC samples occurred at least once for every 10 samples injected to evaluate the stability of the LC-MS method.

#### 2.5.2. UHPLC-QTOF/MS Conditions

The assay was performed with an Agilent 1290 high-performance liquid chromatography system combined with an Agilent 6550 QTOF MS (Agilent Technologies, CA, USA).

Chromatographic conditions: The chromatography was performed on a Waters ACQUITY UPLC HSS T3 column (Milford, MA, USA) (100 mm × 2.1 mm, 1.8 μm) at a temperature of 35 °C. The mobile phase was 0.1% formic acid and water (A) and acetonitrile (B), and the flow rate was 0.3 mL/min. Gradient elution: 0–5 min, 5–45% B; 5–8 min, 45–80% B; 8–10 min, 80–95% B; and 10–15 min, 95% B. Post time: 5 min. The injection volume was 10 μL in positive ion mode and 5 μL in negative ion mode.

Mass spectrum conditions: Positive ion mode: dry temperature: 225 °C; drying gas flow rate: 15 L/min; spray gas pressure: 35 psi; sheath temperature: 350 °C, sheath gas flow rate: 11 L/min; nozzle voltage: 1500 V; capillary outlet voltage: 380 V; capillary voltage: 4000 V (ESI+); mass spectrum scanning range: 50~1200 *m*/*z*. Negative ion mode: dry temperature: 225 °C; drying gas flow rate: 15 L/min; spray gas pressure: 35 psi; heath temperature: 350 °C, sheath gas flow rate: 11 L/min; nozzle voltage: 1500 V; capillary outlet voltage: 380 V; capillary voltage: 3500 V (ESI−); mass spectrum scanning range: 50~1400 *m*/*z*.

#### 2.5.3. Statistical Analysis

The collected UHPLC-QTOF/MS data were imported into Profinder, and the peak discovery, alignment, filtering and normalization of the spectrum were performed. CEF files and CSV files were exported. The Agilent Mass Profiler Professional (MPP, version 15.1) software was used to process the exported CEF file data and compare the differences between the model group and the control group, and between the LD, MD, HD groups and the model group. The different metabolites were obtained by one-way ANOVA, with *p* < 0.05 and fold change > 2 as the screening conditions. At the same time, the SIMCA-P (version 14.0) software was used to perform multivariate statistical analysis on the exported CSV file data. This included unsupervised mode principal component analysis and orthogonal partial least squares discriminant analysis, and VIP values were obtained. Differential metabolites satisfying VIP > 1 and |P(corr)| ≥ 0.58 were further screened as potential biomarkers.

The ID Browser in the MPP software (version 15.1) was used to identify compounds for potential biomarkers, according to the high-resolution mass spectrometry data combined with a database (such as Metlin (http://metlin.scripps.edu, accessed on 5 June 2023) or HMDB (http://www.hmdb.ca, accessed on 7 June 2023), etc.), which were used to identify the compounds. This was matched to obtain information such as the names of potential biomarkers and KEGG ID. Using the MetaboAnalyst 5.0 (https://www.metaboanalyst.ca/, accessed on 10 June 2023) platform for potential biomarkers for screening enrichment, MII’s possible metabolic pathways to protect the liver were obtained.

IBM SPSS Statistics (version 26) was used for the statistical analysis of the results of biochemical indicators. The mean ± SD was used to represent the content and deviation, and an independent samples *t*-test was used for comparison between the groups. *p* < 0.05 represented a significant difference.

### 2.6. Network Pharmacology

#### 2.6.1. Drug Target Prediction

The Pubchem (http://pubchem.ncbi.nlm.nih.gov/, accessed on 12 June 2023) website was used to obtain the MII structure result with the keyword set to “magnesium isoglycyrrhizinate”, and the SDF format file was downloaded. Using the Swiss Target Prediction website (http://swisstargetprediction.ch/, accessed on 12 June 2023) and PharmMapper (http://lilab-ecust.cn/pharmmapper, accessed on 12 June 2023), the target prediction of magnesium isoglycyrrhizinate was carried out, and Homo sapiens was selected as the species. The results were combined, and then unified correction was carried out using the Uniprot database (https://www.uniprot.org/, accessed on 13 June 2023) as the target of MII.

#### 2.6.2. Disease Target Collection

“APAP-induced liver damage”, “drug-induced liver injury”, “APAP-induced liver injury”, “drug-induced liver damage” and “liver injury” were used as keywords to search the GeneCards database (https://www.genecards.org/, accessed on 13 June 2023) and OMIM database (https://www.omim.org/, accessed on 13 June 2023), in order to retrieve the disease-related targets, and the results were pooled. Then, the intersection of MII drug targets and disease targets was carried out.

#### 2.6.3. Construction and Analysis of Protein Target Interaction PPI Network

The intersection genes obtained in the above steps were imported into the String website (http://string-db.org, accessed on 13 June 2023), the genus was set as “Homo sapiens”, and the interaction score was set as ≥0.4. TSV result files of protein interactions were retrieved and downloaded. The topological attribute analysis of the PPI network was performed using the Cytoscape 3.7.2 software, and the plug-in “cytoHubba” was used to analyze the contribution of targets, screen the top 10 “hub genes” and identify the core targets.

#### 2.6.4. GO and KEGG Path Analysis

The intersection genes were added to the DAVID database (https://david.ncifcrf.gov/, accessed on 15 June 2023); the species was set to “Homo sapiens”, the “list type” was set to “gene list”, and the identifier was set to the official gene symbol. The GO biological function analysis included three parameters: biological process (BP), cellular component (CC) and molecular function (MF); the KEGG pathway significance level was set to *p* < 0.05. Based on the results, the Gene Ontology (GO) biological function analysis and Kyoto Encyclopedia of Genes and Genomes (KEGG) pathway analysis were conducted for the intersection targets. Using the microscopic letter online platform (http://www.bioinformatics.com.cn/, accessed on 15 June 2023), a GO function analysis histogram was constructed and KEGG pathway analysis enrichment of the bubble chart was carried out.

#### 2.6.5. Molecular Docking

The targeted compound MII was docked with target proteins (JAK2 and STAT3) through the SYBYL-X software (version 2.0) to verify the potential mechanism. The threshold was set as 5 [28]. Through this method, the results of network pharmacology were verified.

### 2.7. Protein Expression Was Verified by Western Blot Analysis

A certain amount of liver tissue from the mice in each group was ground with liquid nitrogen, a lysate was added, and this was centrifuged at 12,000 rpm at 4 °C for 20 min, to extract the protein from the supernatant. The protein mass concentration was determined using the BCA method. Using GADPH as the internal reference antibody, electrophoresis was performed on the upper sample hole of the rubber plate, the adhesive block of the target protein strip was cut and transferred to a PVDF membrane, and 5% skim milk powder was added to the seal for 2 h. Then, JAK2 and STAT3 antibodies were added, before being incubated overnight. The PVDF membrane was rinsed 3 times and incubated with sheep anti-rabbit IgG antibody, labeled by HRP. The developer and fixer were prepared according to the development and fixer kit manufacturers’ instructions. The film was scanned, and the gray values of the target strip were analyzed by the ImageJ software (1.48v).

## 3. Results

### 3.1. Effects of MII on ALT, AST and SOD Levels in the Serum of Mice with Liver Injury

**Figure 1:**
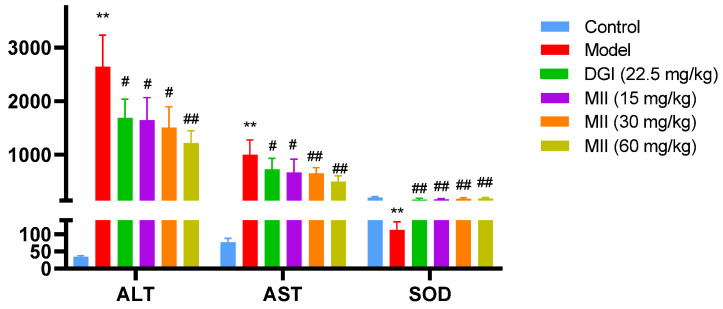
Effects of MII on biochemical indices of mice with liver injury (** compared with control group, *p* < 0.01; # compared with the model group, *p* < 0.05; ## compared with model group, *p* < 0.01).

ALT and AST were significantly increased in the model group compared with the control group. After MII administration, the above indicators in the low-, medium- and high-dose (LD, MD and HD) groups decreased significantly compared with the model group (Figure 1). The trend in SOD was contrary to the above indicators: compared with the control group, SOD activity in the model group was significantly reduced; after MII administration, SOD activity in the low-, medium- and high-dose groups was higher than that in the model group (Figure 1).

### 3.2. Effect of MII on Liver Histopathology in Mice with Liver Injury

**Figure 2:**
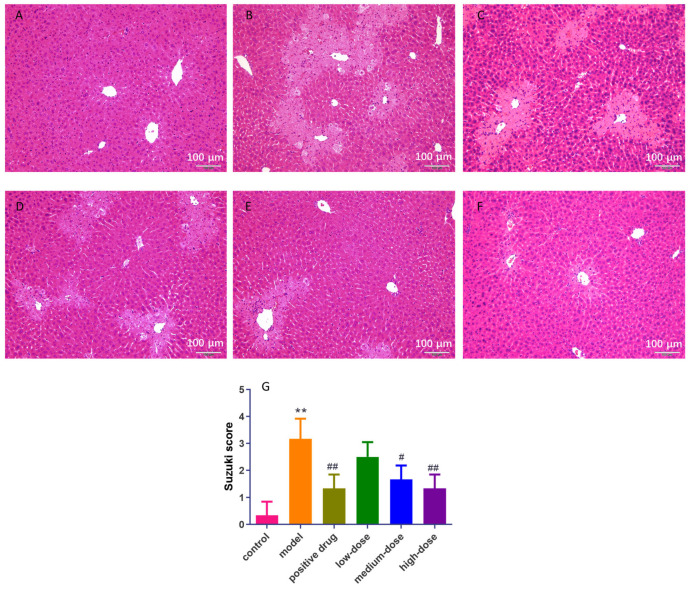
Effect of MII on pathological changes in liver HE staining in mice with liver injury (magnification 200 times). (**A**) Control group; (**B**) model group; (**C**) positive drug group; (**D**) low-dose group; (**E**) medium-dose group; (**F**) high-dose group; (**G**) Suzuki score on each group (** compared with control group, *p* < 0.01; # compared with the model group, *p* < 0.05; ## compared with model group, *p* < 0.01).

The histopathological results are shown in Figure 2. In the control group, no obvious abnormalities were observed. The structure of hepatic lobules was clear. Hepatocyte degeneration and necrosis were not observed, and inflammatory cell infiltration in the conjunctival area and small bile duct hyperplasia were not observed (Figure 2A). In the model group, the liver cells around the sink area were necrotic and the staining was light. The necrotic areas were connected into slices. In addition, hepatic sinus stenosis or disappearance and inflammatory cells infiltrating the sink area were observed, and some of the sink area had timid duct necrosis (Figure 2B). Compared with the model group, the positive drug group showed some improvement. There was some hepatocyte necrosis around the sink area, and there was no connection between the necrotic areas. Partial hepatic sinus stenosis was observed, and inflammatory cell infiltration was seen in some of the sinks (Figure 2C). In the low-dose group, some hepatocytes around the sink area were necrotic, and the necrotic areas were not connected into sheets. Partial hepatic sinus stenosis was observed and inflammatory cell infiltration was seen in a small part of the portal area (Figure 2D). A small number of hepatocytes in the medium-dose group were necrotic. The necrotic areas in the lower-dose group were significantly reduced and no slices were connected. Partial hepatic sinus stenosis was observed and a small amount of inflammatory cell infiltration could be seen in a few of the portal areas (Figure 2E). In the high-dose group, necrosis of a few hepatocytes occurred around the sink area, and the necrotic area was small. A small amount of hepatic sinus stenosis was observed and a small amount of inflammatory cell infiltration could be seen in a small number of portal areas (Figure 2F). Compared with the model group, the low-dose group and the high-dose group, the Suzuki score was significantly lower (*p* < 0.05, Figure 2G).

The results of histopathological and biochemical indices showed that the mouse liver injury model was successfully prepared, MII administration had a significant liver protection effect, and the improvement effect of the high-dose group was more obvious than that of the low-dose group. Overall, the MII group had a better effect than the DHI group, and the high-dose-MII group (60 mg/kg) had the best effect.

### 3.3. Metabolomics Analysis

#### 3.3.1. Multivariate Statistical Analysis

**Figure 3:**
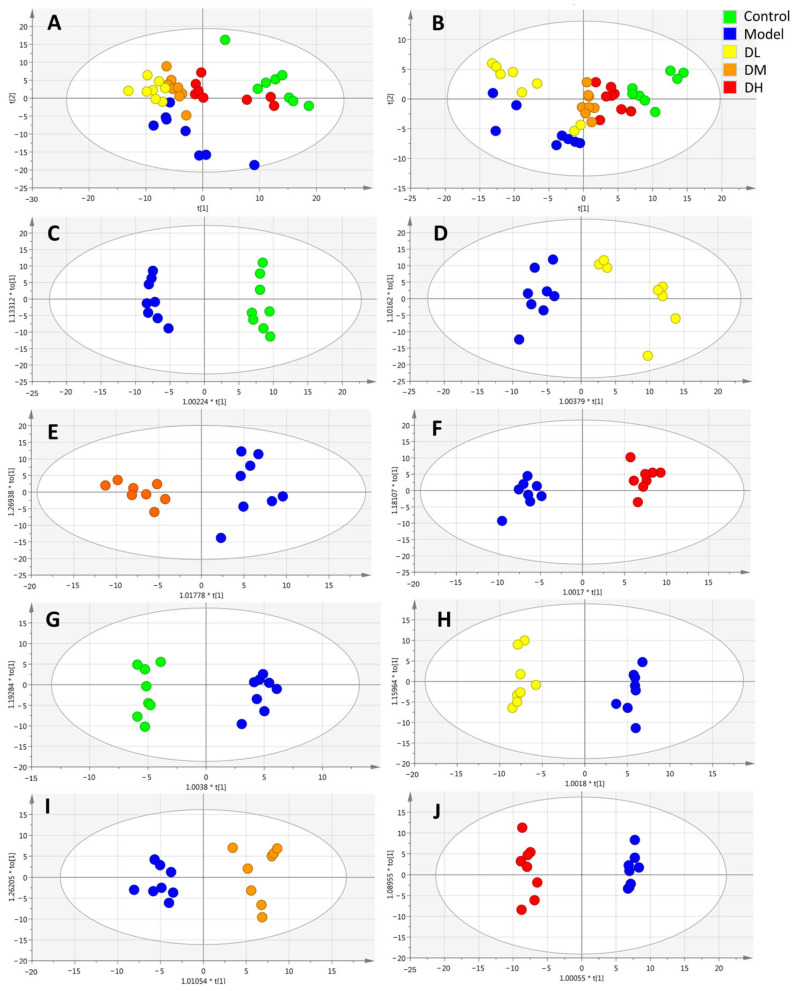
Multivariate statistical analysis of plasma metabolites in mice (PCA scores of positive and negative ion modes of (**A**,**B**); OPLS-DA scores of each group and model group in (**C**–**F**) cationic mode; OPLS-DA scores of (**G**–**J**) negative ion model groups and model groups).

The spectrum was processed by Profinder, and 5081 ions were obtained. All ion data were exported to a CSV file and imported into the SIMCA-P software (version 14.0). Unsupervised PCA was used to perform the overall metabolic profile analysis for each group, reflecting the original state of the data. The results showed that the model group and the control group could be significantly separated under positive and negative ion modes, and the low-, medium- and high-dose groups could be significantly separated from the model group after MII administration (Figure 3A,B). Immediate modeling disturbed endogenous metabolites in mice, and the administration of MII mitigated this change. OPLS-DA was further used to conduct a supervised analysis and comparison between the two groups, and it was found that the model group and control group, the low-dose group and model group, the medium-dose group and model group and the high-dose group and model group could be effectively separated under the positive and negative ion modes (Figure 3C–H). The OPLS-DA model uses *R*^2^*X*, *R*^2^*Y* and *Q*^2^ to evaluate the reliability and analyze whether there is overfitting. In the positive ion mode, the above indices of the model group and control group, the low-dose group and model group, the medium-dose group and model group and the high-dose group and model group were as follows. Positive mode: *R*^2^*X* = 0.449, *R*^2^*Y* = 0.984, *Q*^2^ = 0.855; *R*^2^*X* = 0.386, *R*^2^*Y* = 0.854, *Q*^2^ = 0.875; *R*^2^*X* = 0.255, *R*^2^*Y* = 0.919, *Q*^2^ = 0.666; *R*^2^*X* = 0.497, *R*^2^*Y* = 0.977 and *Q*^2^ = 0.822. Negative mode: *R*^2^*X* = 0.554, *R*^2^*Y* = 0.980, *Q*^2^ = 0.611; *R*^2^*X* = 0.505, *R*^2^*Y* = 0.973, *Q*^2^ = 0.847; *R*^2^*X* = 0.329, *R*^2^*Y* = 0.901, *Q*^2^ = 0.690; *R*^2^*X* = 0.568, *R*^2^*Y* = 0.990 and *Q*^2^ = 0.957.

**Figure 4:**
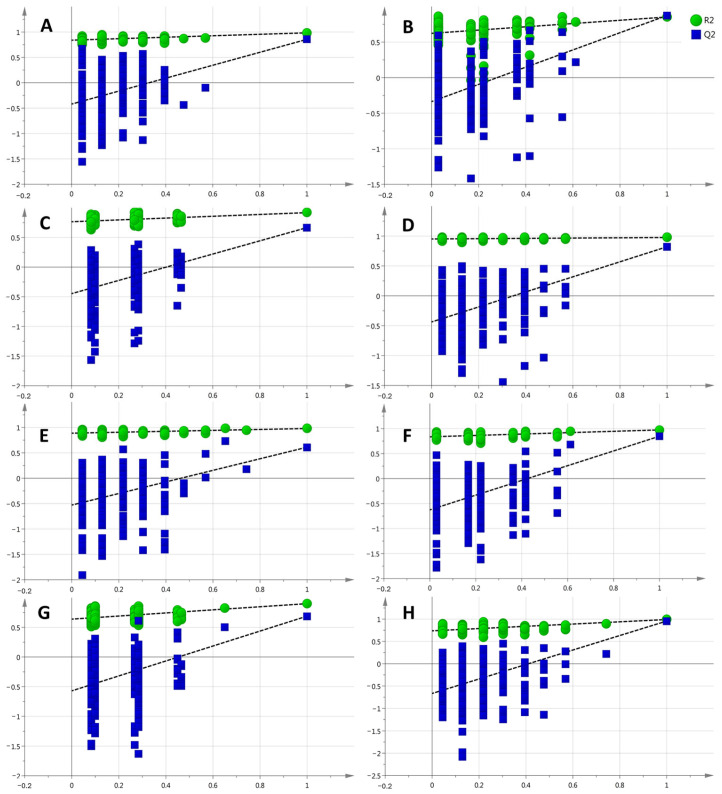
Results of OPLS-DA replacement validation in different groups ((**A**–**D**) positive ion mode control and low-, medium- and high-dose groups and model group, respectively; OPLS-DA replacement verification results; verification results of OPLS-DA replacement between control, low-dose, medium-dose and high-dose (**E**–**H**) negative ion mode groups and model group, respectively).

The displacement test was used to verify the OPLS-DA model. The results obtained after 200 displacement tests are shown in Figure 4: all of the simulated values were lower than the true value on the far right, and the intercept of the regression line of *Q*^2^ was less than 0.05. The above results show that the model had a good fitting degree and prediction ability, and there was no overfitting phenomenon.

#### 3.3.2. Analysis of Method Stability

**Figure 5:**
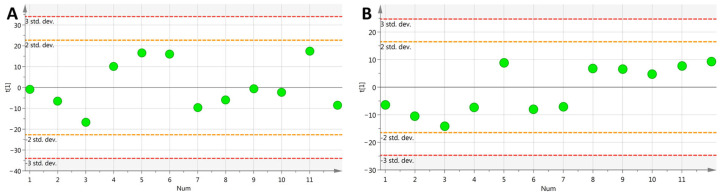
PCA scores of QC samples ((**A**) positive ion mode; (**B**) negative ion mode).

Unsupervised PCA was used to analyze QC samples, and it was found that all QC samples were within the range of two times the standard deviation (as shown in Figure 5). The results show that the method had good stability and repeatability, and the data obtained in this experiment were stable and reliable.

#### 3.3.3. Potential Biomarker Screening

In order to obtain biomarkers related to the mechanism of MII liver protection, potential biomarkers were screened according to the methods described in Section 2.5.3 (*p* < 0.05, fold change > 2, VIP > 1, and |P(corr)| ≥ 0.58). A variety of software and databases were used to match metabolites, and potential biomarkers were identified comprehensively by combining high-resolution data, secondary mass spectrometry data and the literature information. A total of 29 potential biomarkers were screened, and their specific information is shown in Table 1 (with the total ion chromatogram (TIC) in Supplementary Materials Figure S1). Compared with the control group, the plasma levels of sphinganine, glutamate, LysoPC (18:1), PC (16:0/16:0) and methacholine were significantly increased in liver injury model mice, and the administration of MII significantly reduced the levels of these metabolites. The levels of cytosine, L-cystine, L-methionine, L-valine and sphingosine 1-phosphate were significantly reduced, and MII could significantly regulate these metabolites.

#### 3.3.4. Metabolic Pathway Analysis

**Figure 6:**
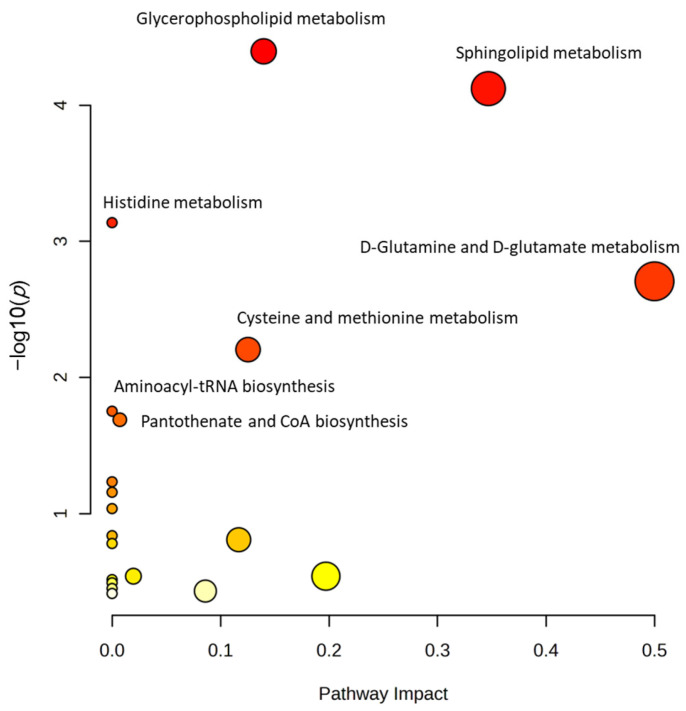
Enrichment of potential biomarker metabolic pathways.

The 29 screened potential biomarkers were introduced into the MetaboAnalyst platform for the enrichment of metabolic pathways. Seven metabolic pathways that were closely related to the liver protective mechanism of MII were obtained using an impact value > 0.1 and significance level of *p* < 0.05 as the criteria for screening. The results are shown in Table 2. These pathways include glycerophospholipid metabolism, sphingolipid metabolism, histidine metabolism, glutamine and glutamate metabolism, cysteine and methionine metabolism, aminoacyl biosynthesis and pantothenic acid and coenzyme A biosynthesis, etc. (Figure 6). It has been suggested that the protective effect of MII on the liver may be related to the regulation of lipid metabolism (such as glycerophospholipids, sphingolipids) and amino acid metabolism (such as histidine, glutamic acid, cysteine and methionine).

### 3.4. Network Pharmacological Analysis

#### 3.4.1. Analysis of Targets of MII and Drug-Induced Liver Injury

According to the method described in Section 2.6, a total of 395 MII action targets and 1296 drug-induced liver injury disease targets were obtained. A total of 44 possible targets of MII in the treatment of drug-induced liver injury were selected.

#### 3.4.2. Construction of PPI Network in MII Treatment of Liver Injury

After the 44 intersection targets were imported into the String database, the PPI network was obtained. The PPI action network involved 44 nodes with 484 edges, and the average node degree value was 22.

#### 3.4.3. Analysis of Key Targets of MII in the Treatment of Liver Injury

**Figure 7:**
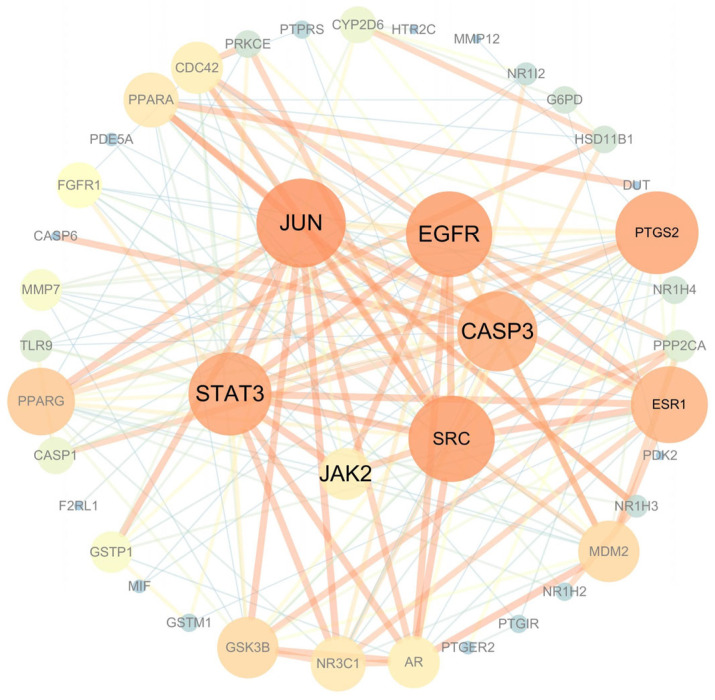
Network analysis diagram of key targets.

According to the network topology parameters, the Cytoscape software (version 3.7.2) uses the “cytoHubba” plug-in to screen drug disease intersection targets. The “hub gene” is a gene that plays an important role in biological processes, regulating other genes in a dominant pathway. The top six key targets of MII in the treatment of liver injury were STAT3, JAK2, JUN, EGFR, CASP3 and SRC. The larger the node, the more important it is, as shown in Figure 7.

#### 3.4.4. Analysis of GO and KEGG Enrichment Results in MII Treatment of Liver Injury

**Figure 8:**
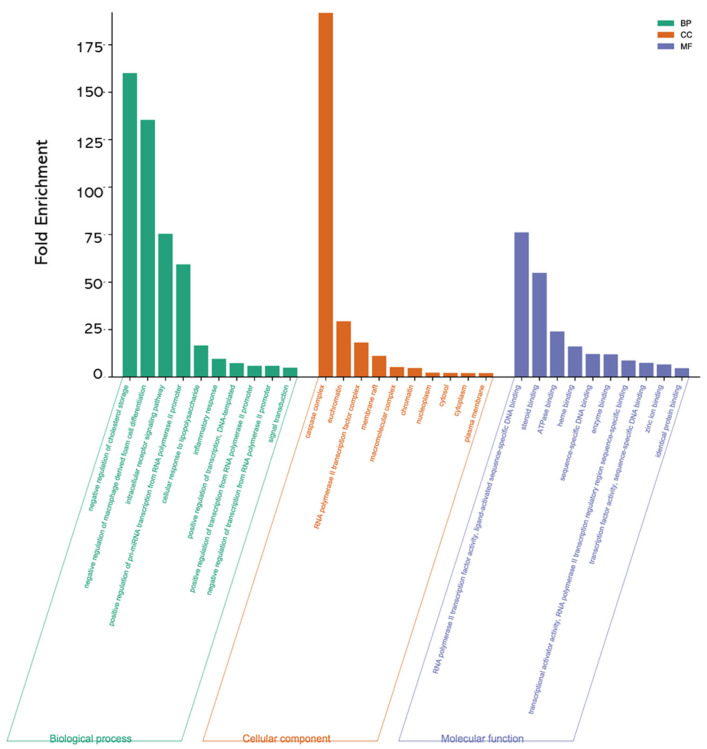
Histogram of GO enrichment analysis results.

A total of 44 intersection targets were selected for GO enrichment analysis through the DAVID database, and a total of 430 items were obtained, including 213 BP, 25 CC and 59 MF. The top 10 items were selected according to the significance level of *p* < 0.05. BP is mainly involved in the negative regulation of cholesterol storage and the negative regulation of “macrophage-derived foam cell differentiation”, the intracellular receptor signaling pathway, etc. CC mainly involves the caspase complex, euchromatin, RNA polymerase II transcription factor complex, etc. MF is mainly involved in RNA polymerase II transcription factor activity, ligand-activated sequence-specific DNA binding, steroid binding and ATPase binding, as shown in Figure 8.

**Figure 9:**
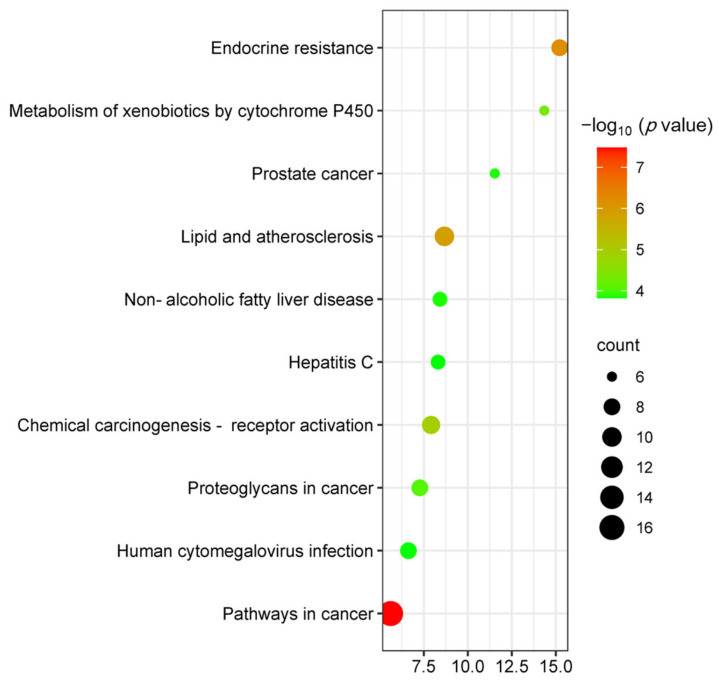
KEGG pathway analysis bubble map.

KEGG analysis showed that the targets of MII treatment of liver injury were mainly enriched in 67 signaling pathways (*p* < 0.05), and the top 10 KEGG pathways were selected according to their *p*-values. Important roles are pathways in cancer, endocrine resistance, lipid regulation and atherosclerosis and chemical carcinogenicity—receptor activation, etc.—as shown in Figure 9.

#### 3.4.5. Molecular Docking

**Figure 10:**
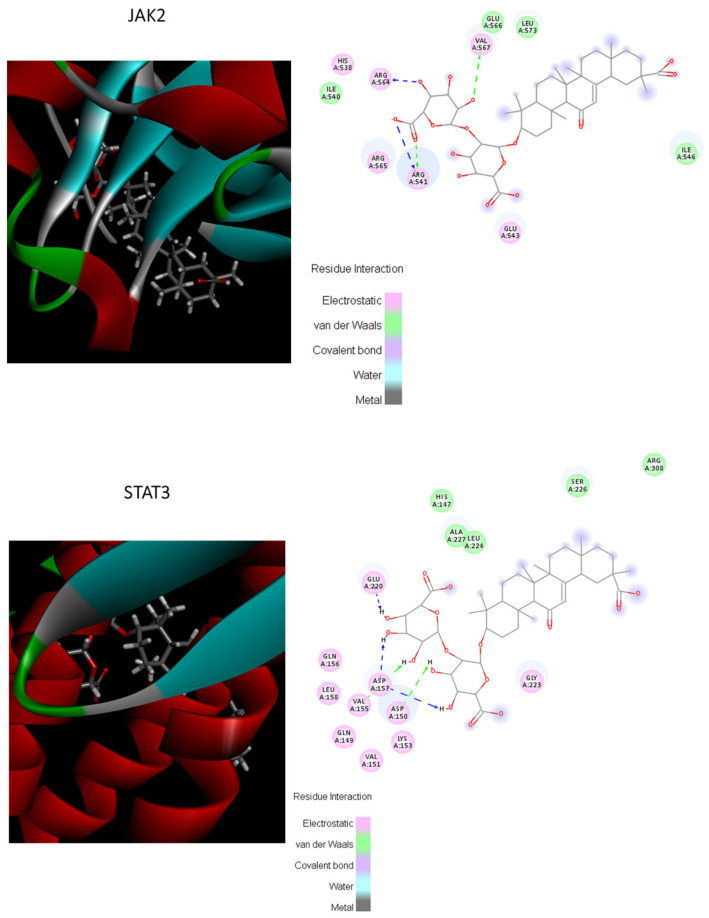
Visualization of the binding of hemostatic components to coagulation target.

The potential bonding possibilities of MII and the proteins were assessed by the molecular docking score, based on the theory that a higher score usually represents a greater bonding possibility; meanwhile, a higher total score means more stable ligand–target binding. The total score of JAK2 and STAT3 was 5.0982 and 5.579, respectively. These results of molecular docking showed that JAK2 and STAT3 were well bound with MII, with a total score above 5 (as shown in Figure 10).

### 3.5. Effects of MII on the Expression of JAK2 and STAT3 in the Liver Tissue of Mice with Liver Injury

**Figure 11:**
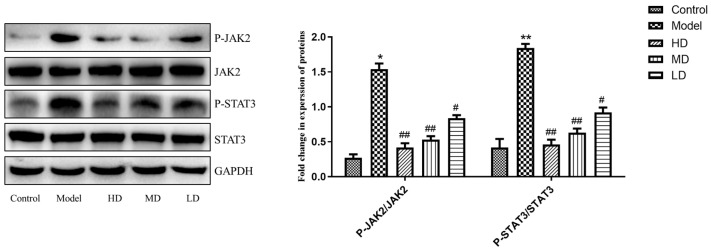
Effect of MII on the expression of JAK2 and STAT3 signal-pathway-related proteins in APAP-induced acute liver injury in mice. (* compared with control group, *p* < 0.05; ** compared with control group, *p* < 0.01; # compared with the model group, *p* < 0.05; ## compared with model group, *p* < 0.01).

In order to verify the above key enzymes, Western blot analysis was performed (as shown in Figure 11). In the model group, the expression of p-JAK2/JAK2 and p-STAT3/STAT3 was significantly increased, and the MII low-dose, middle-dose and high-dose groups could significantly reduce the expression of p-JAK2/JAK2 and p-STAT3/STAT3 (*p* < 0.05); the high-dose group was the most significant (*p* < 0.01).

## 4. Discussion

Metabolomics techniques can collect endogenous compound information from batch samples in a short period of time, especially using the high sensitivity and resolution of high-resolution mass spectrometry to assist in the discovery and identification of metabolites. Current metabolomic analysis strategies are widely used in disease and drug mechanism research and biomarker development [29,30]. Network pharmacology has been used to understand the relationships between drugs and organisms from the perspective of improving, restoring or rebuilding the network balance of systems and discovering possible targets and pathways [31]. In this study, we found that MII may play a role in protecting the liver by regulating lipid-metabolism-related pathways through combined metabolomics and network pharmacology, and its mechanism may be related to down-regulating the expression of two proteins, JAK2 and STAT3, which occurred via the lipid metabolism pathway.

APAP is widely used clinically as an antipyretic and analgesic agent, and an overdose of APAP can cause typical drug-induced liver injury [7]. APAP-induced models of liver injury are commonly used to evaluate the efficacy of hepatoprotective drugs [32]. For example, Zeeyauddin, K. et al. reported that Boswellia serrata leaf extracts showed hepatoprotective activity in APAP-induced liver injury in rats [33]. APAP is metabolized by the liver, and it could produce a toxic intermediate, benzoquinone imide. If it cannot be combined with glutathione in time and excreted from the body, it will cause the intermediate to bind to proteins in the liver, leading to cell necrosis and resulting in elevated serum ALT and AST. In this study, the content of the above markers in the model group increased significantly, reflecting the damage caused to the livers of model mice; this was consistent with previous results [34]. The content of the above markers decreased significantly after the administration of MII, indicating that MII had a hepatoprotective effect on liver-damaged mice. SOD is an active oxygen species scavenger that converts superoxide anions into hydrogen peroxide in the body, and then converts them into water through other enzymes. SOD can reflect the degree of oxidative stress that the body is subjected to. In this study, SOD activity decreased after modeling, indicating oxidative stress damage in the model group mice, which was consistent with the results reported in the literature [35]. The activity of SOD was significantly increased after administration, indicating that MII can effectively reduce oxidative stress, thereby playing a role in protecting the liver [36].

The liver is an important metabolic organ and is also an important place for amino acid synthesis and metabolism. Abnormal levels of amino acids indicate the damage caused by the livers of model mice [37,38]. Glutamate is a major excitatory amino acid involved in the coordinated regulation of energy metabolism, glutamine synthesis and ammonia detoxification [39]. The content of glutamate is significantly increased in the disease state of liver injury [40], and MII can reverse this trend, exert its regulatory role in amino acid metabolism and then protect the liver. Methionine can be used as a precursor for glutathione synthesis and is the main methyl donor for nucleic acids, phospholipids, histones, biogenic amines and protein methylation. Metabolic disorders involving methionine can lead to pathological damage to the liver, and methionine supplementation helps to reduce liver damage [41]. In this study, the administration of MII significantly upregulated the methionine content. Based on the above, MII may exert a hepatoprotective effect by regulating related amino acid metabolism pathways.

Lipid changes are the causes and consequences of many liver diseases, and the dynamic changes in glycerophospholipids, phospholipids, sphingolipids and ceramides are closely related to the occurrence and development of liver diseases [42]. Glycerophospholipids are the most abundant and complex phospholipids in living organisms and are also important components of liver cell membranes and mitochondrial membranes [43]. An increase in phosphatidylcholine (PC) content after modeling indicates damage to the mitochondrial membrane and the disturbance of metabolic processes in the body associated with PC [44]. Sphingolipids are widely present in various organisms and are the main components of cell membranes. They are also important factors for a variety of signaling types in cells, participating in cell proliferation, differentiation, gene expression and apoptosis [45]. Studies have also shown that the accumulation of intracellular ceramides is an important factor in inducing the apoptosis of liver cells and causing organ dysfunction [46]. Sphingosine can induce apoptosis by reducing cell contractility, and phytosphingosine can induce apoptosis by activating caspase-3 and releasing cytochromes [47]. The content of dihydroceramide and phytoceramide increased significantly after modeling, indicating that the sphingolipid metabolism of rats in the model group was disordered, and acetaminophen may induce apoptosis through the above mechanism, causing liver dysfunction. The administration of MII significantly alleviated the above metabolic abnormalities, improved sphingolipid metabolism, and played a role in protecting the liver.

STAT3 can directly or indirectly regulate the expression of important genes in the process of liver repair, becoming an essential regulatory factor in this process. It is mainly coupled with the JAK family of tyrosine protein kinases in the intracellular matrix, and it participates in the signaling process of interleukin and other extracellular signaling molecules in liver cells in the form of the JAK/STAT3 pathway. It also has significant anti-apoptosis and pro-mitosis activity in relation to STAT3 [48].

In summary, 29 potential biomarkers related to liver injury and MII’s therapeutic effects were screened and enriched in seven metabolic pathways. Combined with network pharmacological analysis, we have shown that the mechanism of MII in protecting the liver may be achieved by regulating lipid metabolism and amino-acid-metabolism-related pathways Western blot analysis verification confirmed that the mechanism of action of MII may be related to the significant downregulation of the expression of JAK2 and STAT3. This study provides scientific data for the study of the mechanism of action of MII in the prevention and treatment of liver injury from the perspective of metabolomics and network pharmacology. Moreover, it can provide a reference for the rational clinical application of MII.

## 5. Conclusions

In this study, mice with acetaminophen-induced liver injury were used as the research object to explore the mechanism of action of MII to protect the liver. The modeling situation and MII efficacy were evaluated by histopathology and biochemical index analysis. The metabolomic analysis of mouse plasma was carried out by UHPLC-QTOF/MS technology, and potential biomarkers were screened, metabolic pathways were enriched, and their mechanism of action was explored. In this study, a total of 29 potential biomarkers were identified that were closely related to liver injury and the liver protective effect of MII, and the administration of MII could significantly recall these potential biomarkers. Metabolic pathway analysis showed that MII mainly works by regulating lipid metabolism (such as glycerophospholipids, sphingolipids) and amino acid metabolism (such as histidine, glutamic acid, cysteine and methionine). Network pharmacology yielded 44 intersection targets for MII in the treatment of liver injury, involving lipid metabolism and other related pathways. Western blot analysis results showed that MII could significantly reduce the expression of JAK2 and STAT3.

## Figures and Tables

**Table 1:** Potential biomarkers associated with liver protection by MII.

No.	Match	RT (min)	*m*/*z*	Formula	Scan Mode	VIP	*p*	Model vs. Control	Dose vs. Model
1	2,5-Dichloro-4-oxohex-2-enedioate	1.28	226.9509	C_6_H_4_Cl_2_O_5_	Positive	3.7	0.0000913	up	down
2	Glutamate	1.32	148.0604	C_5_H_9_NO_4_	Positive	2.4	0.00126	up	down
3	β-Guanidinopropionic acid	1.49	130.0622	C_4_H_9_N_3_O_2_	Negative	2.2	3.76 × 10^−10^	up	down
4	γ-Glutamyl-β-cyanoalanine	1.56	244.0928	C_9_H_13_N_3_O_5_	Positive	4.3	0.000124	up	down
5	Methacholine	1.62	161.1410	C_8_H_18_NO_2_	Positive	2.0	1.41 × 10^−6^	up	down
6	L-Valine	1.68	280.1391	C_11_H_21_NO_7_	Positive	2.4	0.00155	down	up
7	L-Methionine	1.87	150.0583	C_5_H_11_NO_2_S	Positive	1.1	0.00395	down	up
8	Pyrrolidonecarboxylic acid	1.95	130.0499	C_5_H_7_NO_3_	Positive	2.6	0.00776	down	up
9	Cytosine	2.01	112.0505	C_4_H_5_N_3_O	Positive	5.1	0.0285	down	up
10	3,5-Dihydroxybenzoic acid	2.12	121.0295	C_7_H_6_O_2_	Negative	3.6	0.0000357	down	up
11	2-Phenylacetamide	2.20	136.0757	C_8_H_9_NO	Positive	3.9	6.12 × 10^−12^	down	up
12	Aminocaproic acid	2.32	130.0874	C_6_H_13_NO_2_	Negative	3.1	0.00126	down	up
13	1-Acyl-sn-glycero-3-phosphoserine	3.25	288.0479	C_7_H_14_NO_9_P	Positive	2.5	0.000773	down	up
14	Imidazoleacetic acid	3.41	125.0357	C_5_H_6_N_2_O_2_	Negative	2.3	0.00051	down	up
15	Citicoline	3.58	513.1122	C_14_H_27_N_4_NaO_11_P_2_	Positive	4.4	0.0134	down	up
16	Pantothenic acid	3.67	218.1034	C_9_H_17_NO_5_	Negative	2.2	9.90 × 10^−5^	down	up
17	5-Methylthioadenosine	3.94	298.0968	C_11_H_15_N_5_O_3_S	Positive	1.4	3.27 × 10^−6^	up	down
18	5-Methylthiopentanaldoxime	4.12	148.0791	C_6_H_13_NOS	Positive	2.2	0.000336	down	up
19	1-Methylhistidine	4.31	170.0924	C_7_H_11_N_3_O_2_	Positive	2.7	1.23 × 10^−6^	down	up
20	L-Cystine	5.22	209.0591	C_6_H_12_N_2_O_4_S	Positive	4.8	2.23 × 10^−4^	down	up
21	Sphinganine	7.85	302.3054	C_18_H_39_NO_2_	Positive	3.2	3.61 × 10^−5^	up	down
22	Phytosphingosine	7.91	318.3003	C_18_H_39_NO_3_	Positive	3.2	1.39 × 10^−5^	up	down
23	Dihydroceramide (d18:0/18:0)	8.51	568.5663	C_36_H_73_NO_3_	Positive	1.6	6.72 × 10^−3^	down	up
24	Sphingosine 1-phosphate	8.89	380.2560	C_18_H_38_NO_5_P	Positive	2.4	0.0000861	down	up
25	α-Methylstyrene	9.36	119.0855	C_9_H_10_	Positive	3.1	2.11 × 10^−8^	up	down
26	2-Acyl-sn-glycero-3-phosphocholine	9.40	543.3319	C_28_H_49_NO_7_P	Positive	3.5	0.00713	down	up
27	LysoPC (18:1)	9.41	495.3319	C_24_H_49_NO_7_P	Positive	1.8	2.31 × 10^−4^	up	down
28	α-D-galactosyl undecaprenyl diphosphate	9.86	1089.5919	C_61_H_102_O_12_P_2_	Positive	5.3	2.10 × 10^−5^	down	up
29	PC (16:0/16:0)	12.37	734.5694	C_40_H_80_NO_8_P	Positive	5.6	0.001266	up	down

*p* < 0.05, VIP > 1 were the screening conditions.

**Table 2:** Metabolic pathways enriched by potential biomarkers and related information.

Name	Total	Hits	*p*	FDR	Impact
Glycerophospholipid metabolism	36	5	5.36 × 10^−5^	0.0039812	0.13975
Sphingolipid metabolism	21	4	9.48 × 10^−5^	0.0039812	0.34686
Histidine metabolism	16	3	8.61 × 10^−4^	0.024097	0
Glutamine and glutamate metabolism	6	2	0.00220	0.046115	0.5
Cysteine and methionine metabolism	8	2	0.00404	0.067834	0
Aminoacyl biosynthesis	33	3	0.00732	0.10244	0.12535
Biosynthesis of pantothenic acid and coenzyme A	48	3	0.02067	0.23835	0

## Data Availability

The authors declare that all data supporting the findings of this study are available within the article.

## Supplementary Materials

The following supporting information can be downloaded at: https://www.mdpi.com/article/10.3390/cimb46010019/s1, Figure S1: The the total ion chromatogram (TIC) of real samples. The top is TIC in positive ion mode, and the bottom is TIC in negative ion mode.
