# Supplementary material for: Integrating Metabolomics and Network Pharmacology to Decipher the Hepatoprotective Effect Mechanisms of Magnesium Isoglycyrrhizinate Injection
Source: Curr Issues Mol Biol. 2023 Dec 29;46(1):279–98. doi: 10.3390/cimb46010019 (PMC10813909; doi:10.3390/cimb46010019)
Supplement: Supplementary file 1 [file cimb-46-00019-s001.zip › cimb-2757431-supplementary.pdf]

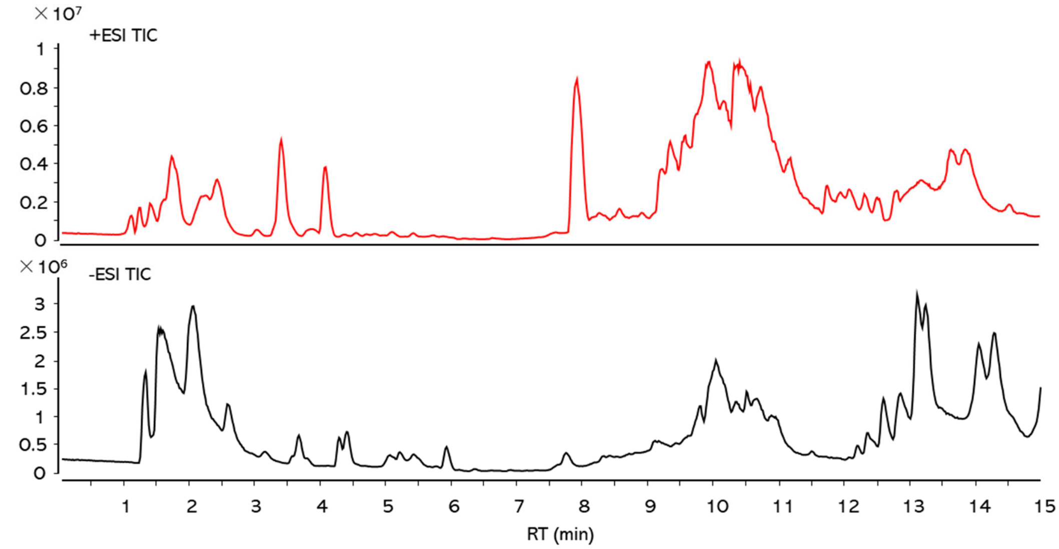

Figure S1. The the total ion chromatogram (TIC) of real samples. The top is TIC in positive ion mode, and the bottom is TIC in negative ion mode.
